# Epiretinal membrane appearance or progression after intravitreal injection in age-related macular degeneration

**DOI:** 10.1186/s12886-021-01944-0

**Published:** 2021-04-28

**Authors:** Hikari Taniguchi, Izumi Yoshida, Masashi Sakamoto, Takatoshi Maeno

**Affiliations:** 1 Sakura Medical Center, Toho University, 285-8741 Sakura-shi, Chiba, Japan; 2 Toho-Kamagaya Hospital, 273-0132 Kamagaya-shi, Chiba, Japan

**Keywords:** Epiretinal membrane, Age-related macular degeneration, Intravitreal injection, Posterior vitreous detachment

## Abstract

**Background:**

The purpose of this study is to evaluate the influence of anti-vascular endothelial growth factor (VEGF) in the appearance or progression of epiretinal membranes (ERMs) in age-related macular degeneration (ARMD) and investigate confounding factors causing ERMs.

**Methods:**

Seventy-six eyes that were treated for more than 36 months from the first anti-VEGF injection were assessed. Binary logistic regression analysis was performed between smoking, lens status, subretinal hemorrhage, posterior vitreous detachment (PVD) status, peripheral retinal degeneration, type of AMD, conditions of contralateral eye, and the number of injections as independent variables and appearance or progression of ERMs during 36 months as dependent variables.

**Results:**

The presence of vitreomacular adhesion (VMA) or development of PVD during the observation period was significantly associated (Odds ratio [OR]: 5.77; 95% confidence interval [CI], 1.72–19.4; *p* = 0.005) with the appearance or progression of ERMs. Moreover, peripheral retinal degeneration was significantly associated (OR: 3.87; 95% CI, 1.15–13.0; *p* = 0.029). Injection number of anti-VEGF was not significantly associated (OR: 1.02; 95% CI, 0.90–1.16; *p* = 0.72).

**Conclusion:**

This study suggests possibilities that anti-VEGF injections alone are unable to cause the development of ERMs, that VMA or developing PVD has a prior impact on the developing ERMs in ARMD similar to that of idiopathic ERMs, and that peripheral retinal degenerations and vitreomacular adhesion were both related to ERMs development and pathogenesis of ARMD.

## Background

Epiretinal membranes (ERMs) are a common type of fibro-cellular proliferations that form a fibrous sheet along the inner retinal surface. The Beaver Dam Study revealed the prevalence in older adults as 11.8% [1] and the Blue Mountains Eye Study (BMES) reported it as 7% [2].

Recently, several articles reported the occurrence of ERMs in patients with age-related macular degeneration (ARMD) [3–5]. Eyes with both ARMD and ERMs require more frequent injections of anti-vascular endothelial growth factor (VEGF) drugs than eyes with ARMD alone, due to the prohibited drug penetration or presence of tractional cystoid changes [3]. However, to the best of our knowledge, no report has investigated the relationship between intravitreal injection and appearance or progression of ERMs in ARMD patients.

A previous study discussed the possible progression of ERM after intravitreal ranibizumab injection in branch retinal vein occlusion (BRVO). Marticorena et al. reported that 4/25 (16%) eyes injected with anti-VEGF drugs progressed ERMs 6–7 weeks after injection nevertheless had been diagnosed 5.5–12.5 months earlier [6]. They suggested that regression of VEGF led to decreased nitric oxide (NO) and could induce hypoxia, resulting in up-regulation of platelet derived growth factor (PDGF)-A, tumor necrosis factor (TNF)-α, transforming growth factor (TGF)-β, and ERMs progression [7–9]. A similar condition could appear after intravitreal injection against ARMD, though no issue in BRVO or ARMD has been investigated further.

Clinicians would not concentrate on ERM appearance or progression in patients with ARMD because those rarely affect the visual outcome. However, the progression of ERMs proliferation is gradual over several years. The purpose of this study was to investigate the relationship between intravitreal injection and progression of ERMs taking into account the confounding factors.

## Methods

The study design was approved by the Ethics Committee of Sakura Medical Center, Toho University (No. S18032). The Institutional Review Board (IRB) of Sakura Medical Center, Toho University and the study design adhered to the tenets of Declaration of Helsinki. All patients provided written informed consent for treatments; all private patient information was excluded from the database. The use of anonymous information was approved by the IRB without the need to seek further consent. In a retrospective manner, medical records of all consecutive patients who had visited the ARMD clinic of our hospital between April and August 2019 were evaluated. The durations between the time point when they had first undergone the intravitreal anti-VEGF injection and the time point following 36 months were examined as observation periods. These periods included the time period between July 2012 and August 2019. The inclusion criteria were as follows: those who had continuous treatment of more than 36 months from the first intravitreal injection and evaluation of spectral-domain (SD) optical coherence tomography (Spectralis OCT: Heidelberg Engineering, Heidelberg, Germany). The exclusion criteria were (1) previous vitrectomy; (2) gas injection; (3) photodynamic therapy; (4) previous laser photocoagulation; (5) presence of retinal break; (6) eyes with other conditions that are known to affect the ERMs, such as retinal vascular disease and uveitis; (7) subjects with only time-domain OCT in the early period. All the patients underwent a complete ophthalmological examination. Classic CNV or occult CNV were diagnosed with well-demarcated choroidal fluorescence in the early phase or irregular elevation of the retinal pigment epithelium without an intensely bright area in the early phase and stippled or granular hyperfluorescence on fluorecenin angiography (FA) [10]. On the other hand PCV was diagnosed with choroidal vessels with a polypoidal structure on indocyanine green angiography (IA) in a corresponding lesion on FA [11], and retinal angiomatous proliferation (RAP) was diagnosed with retinal-retinal anastomosis on FA and hot spot on IA [12]. Following diagnosis, these patients were subjected to a treatment protocol that included a loading dose of 3 intravitreal injections with anti-VEGF agents at 1-month intervals. After the third dose, patients followed a pro re nata regimen or a treat-and-extend regimen as decided by each clinician.

We investigated confounding factors causing ERMs (1) smoking [13, 14]; (2) lens status: phakia, pseudophakia, or undergone cataract surgery during the 36 months [15]; (3) subretinal hemorrhage larger than 4-disc area; (4) posterior vitreous detachment (PVD) status: no PVD, complete PVD, anomalous PVD, or progression of PVD during the 36 months [16]; (5) lattice degeneration or any other peripheral retinal degeneration (i.e. cobblestone, naevus, or congenital hypertrophy of the retinal pigment epithelium [CHRPE]);(6) type of exudative AMD; (7) conditions of the contralateral eye [15]; (8) number of injections; and built binary logistic regression analysis to evaluate the multivariate associations between those independent variables and appearance or progression of ERMs as a dependent variable.

**Fig. 1:**
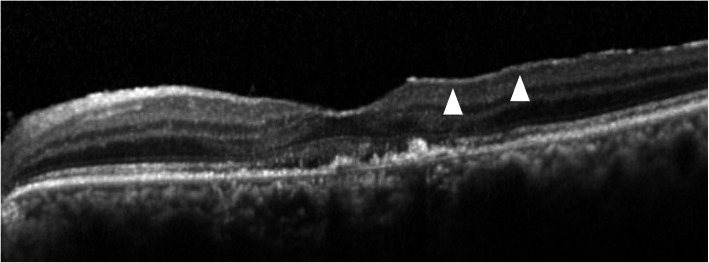
Global adherent (GA). GA is a classification of epiretinal membranes (ERMs) on OCT. GA shows smooth ERMs with no space (arrowheads) above the inner limiting membrane

**Fig. 2:**
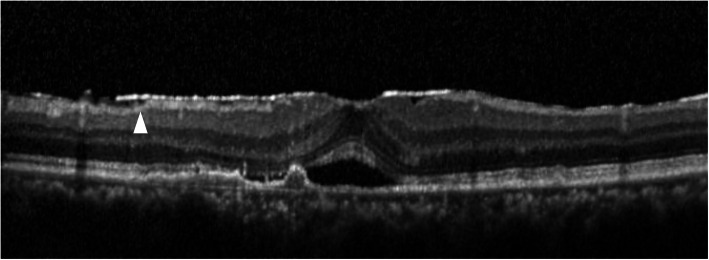
Partial adherent (PA). PA is a classification of epiretinal membranes (ERMs) on OCT. PA shows ERMs with space (arrowhead) above the contracted inner limiting membrane

The classification of ERM was according to previous reports. Kleins et al. classified ERMs into two groups, cellophane macular reflex (CMR) that did not distort the macula, and preretinal macular fibrosis (PMF) that contracted with the appearance of superficial retinal folds, graded by fundus photographs [1]. Wilkins et al. classified membranes into two types, global adherent (or attachment) (GA) (Fig. 1) where no observed area of separation was apparent between the membrane and retinal surface; and partial adherent (or attachment) (PA) (Fig. 2) that had sections separated from the inner limiting membrane observed in OCT [17, 18]. Identifying ERMs with fundus photography is difficult in cases of ARMD because the macula is deformed with choroidal neovascularization, retinal hemorrhage, and fibrotic scar [3]. Hence, we utilized the OCT classification, GA or PA. We used SD-OCT, referred previously, and examined horizontally and vertically over the macular area (consisting of 6 × 6 sq. mm area).

Moreover, ERMs were divided as either idiopathic or secondary, based on etiology following cataract surgery, retinal breaks, laser photocoagulation, retinal cryopexy, ocular inflammation, trauma, vascular abnormalities, and vitreous hemorrhage [19]. Cho et al. reported that 15% of ARMD patients had ERMs. They did not distinguish ERMs in patients with ARMD as idiopathic or secondary [3]. Though ARMD was an inflammatory disease and associated with systemic arterial sclerosis similarly as BRVO and diabetic retinopathy, we regarded ERMs in these patients with ARMD as secondary. Moreover, ERMs after cataract surgery was considered as secondary [19] or idiopathic [18]. Our criteria contained ERMs in ARMD patients with or without cataract surgery, where both were regarded as secondary.

Progression of membranes was defined as per previous reports. Based on fundus photographs, BMESII defined the area of ERM increase more than 25% as an increase and the area of ERM decrease more than 25% as a decrease. Moreover, the proportion of CMR to PMF and disappearance of membranes were evaluated [15]. Byon et al. defined progression as increasing more than 50 μm of central macular thickness, disclosing the distance between superior and inferior arcade vessel, increasing opacities above retina, and modifying GA to PA [18]. We defined appearance or progression of ERMs as no ERMs to appearance of GA or PA, progression from GA to PA because only this distinguishing feature was applied to OCT evaluation. The assessment of OCT images was separately performed by two retinal specialists (H.T and I.Y). If there was any disagreement, they evaluated the data simultaneously and arrived at a consensus.

**Fig. 3:**
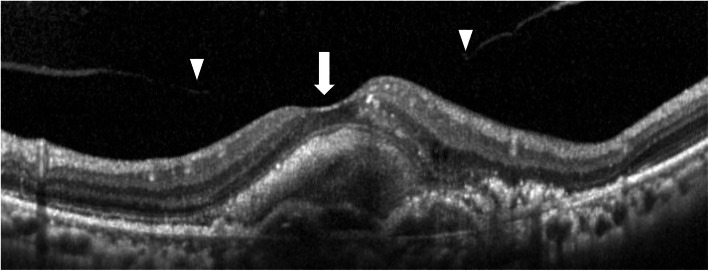
Vitreomacular adhesion (VMA). VMA is a condition of posterior vitreous detachment, in eyes with both vitreal adhesion (large arrow) and separation (arrowheads) inside 6 × 6 mm sq. around the macula

The PVD status was examined throughout the 36 months period evaluated by SD-OCT, slit-lamp examination, and ultrasonography. The classification was according to previous reports that investigated the PVD status in patients with ARMD [20–23]. We classified it into four degrees, no PVD, complete PVD, vitreomacular adhesion (VMA), and progression PVD during 36 months. VMA was defined that eyes had both vitreal adhesion and separation inside 6 × 6 sq. mm area around macula (Fig. 3).

The injection numbers contained both aflibercept (Eylea, Bayer, Germany) and ranibizumab (Lucentis, Novartis, Switzerland). Moreover, we further built another binary logistic regression analysis, containing all referred determining factors, dealing with each injection number of each medicine as different independent variables.

### Statistical analysis

Data analysis was carried out using SPSS software version 25(IBM, Chicago, IL). Relationships between the appearance or progression of ERMs and considerable determining factors were analyzed by binary logistic regression analysis, referred previously. A Pearson correlation was used for the correlation between the injection number of aflibercept and ranibizumab. A *p*-value of < 0.05 was considered significant.

## Results

A total of 222 ARMD patients visited our ARMD clinic during the study period. The inclusion criteria were met by 76 eyes (mean age, 79.8 ± 6.67). The characteristics of these patients were shown in Table 1.

**Table 1:** The characteristics of the subjects

Age	79.8 ± 6.67
Sex	Men 54 Women 22
Type of CNV	Classic 12, Occult20, PCV 38, RAP 1, unknown 5
Baseline logMAR VA	0.41 ± 0.40
After 36 months logMAR VA	0.51 ± 0.44

*CNV* choroidal neovascularization, *PCV* polypoidal choroidal vasculopathy, *RAP* retinal angiomatous proliferation, *log MAR* logarithm of the minimum angle of resolution, *VA* visual acuity

At baseline, 30 eyes (39%) had no ERM, 33 eyes (43%) had GA, and 13 eyes (17%) had PA. After 3 years, 17 eyes (22%) had no ERM, 35 eyes (46%) had GA, and 24 eyes (32%) had PA. The incidence and appearance or progression during the 3 years were shown in Table 2. The weighted kappa coefficient regarding grading ERMs at baseline and following 36 months were 0.8766 and 0.8034 between the two examiners.

**Table 2:** The incidence and appearance or progression of ERMs

	No ERMs throughout 3 years	No ERMs ↓ GA	No ERMs ↓ PA	GA throughout 3 years	GA ↓ PA	PA throughout 3 years	GA ↓ No ERMs	total
Eyes	16	11	3	24	8	13	1	76

*ERMs* epiretinal membranes, *GA* global adherent, *PA* partial adherent

**Fig. 4:**
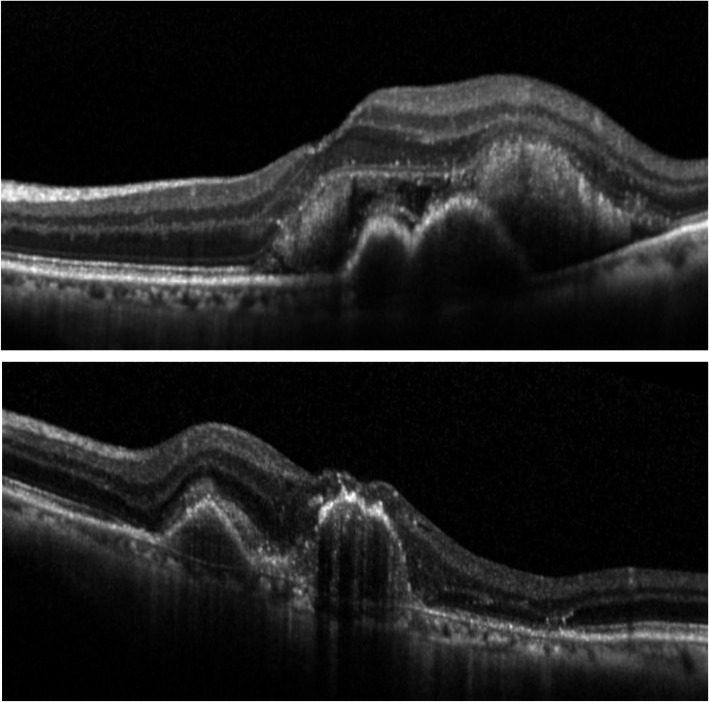
No epiretinal membranes (ERMs) throughout 36 months. A case of an 81–year-old man with polypoidal choroidal vasculopathy. At baseline (top) and at 36 months following the first intravitreal injection (bottom), he presented no ERMs on optical coherence tomography. Aflibercept was injected 6 times and ranibizumab was injected 4 times during this period

**Fig. 5:**
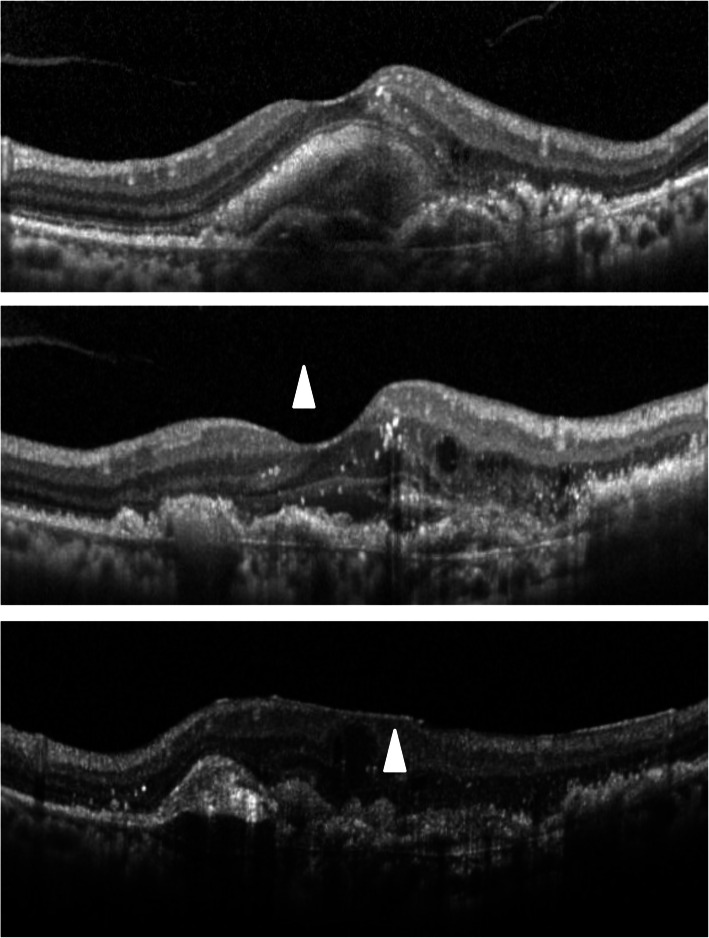
Newly appeared epiretinal membranes (ERMs) in eyes that had no ERMs at baseline. A case of a 76-year-old woman with polypoidal choroidal vasculopathy. At baseline, she presented no ERMs on optical coherence tomography (top). A posterior vitreous detachment (arrowhead) had occurred during the observation period (middle). Following 36 months from the first intravitreal injection, ERMs graded as partial adherent (arrowhead) had appeared (bottom). Aflibercept was injected 16 times and ranibizumab was injected 11 times during this period

**Fig. 6:**
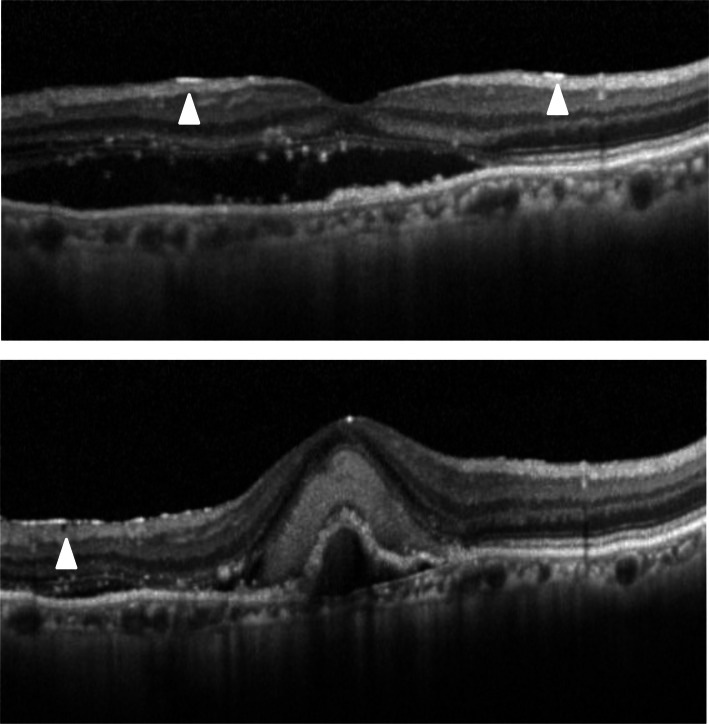
Progression from global adherent (GA) to partial adherent (PA). A case of a 79 –year- old man with occult choroidal neovascularization. At baseline, he already presented with epiretinal membranes (ERMs) identified as GA (arrowheads) on optical coherence tomography (OCT) (top). Following 36 months from the first intravitreal injection, the ERMs had progressed to PA (arrowhead) graded on OCT (bottom). He showed cobblestone degenerations at the peripheral retina and had undergone intravitreal injections with aflibercept 7 times and with ranibizumab 2 times during this period

The representative cases are shown in Fig. 4, 5 and 6.

Table 3 shows the distribution of confounding factors. The results of the binary logistic regression analysis using forced enter method, among these factors as independent variables, showed that presence of VMA or progression of PVD during 36 months against no PVD or complete PVD was significantly associated with ERM appearance or progression. Moreover, the presence of peripheral retinal degeneration was significantly associated as well. Whereas other factors like smoking, cataract surgery, subretinal hemorrhage larger than 4-disc area, type of AMD, prevalence of ERMs in the contralateral eye, and number of injections were not significantly associated. Therefore, we performed the forward-backwards stepwise selection method, and PVD status and peripheral retinal degeneration were also selected as significant variables.

**Table 3:** Multivariate logistic regression analysis about factors that could influence the appearance or progression of ERMs

Variables	Proportion	OR[95% CI]	p
Forced enter method			
**Smoking**	never = 25 (33%) ago = 42 (55%) present = 9 (12%)	0.96 [0.27–3.41]	0.95
**Lens status**	phakia = 52 (68%) pseudophakia = 17 (22%) undergone surgery = 7 (9%)	1.20 [0.31–4.63]	0.80
**Subretinal hemorrhage**	no hemorrhage larger than 4-disc area = 69 (91%) present =7 (9%)	0.44 [0.04–4.85]	0.50
**PVD status**	no PVD = 4 (5%) complete PVD = 46 (61%) VMA = 18 (24%) progress = 8 (11%)	5.77 [1.72–19.4]	0.005
**Peripheral degeneration**	None = 49 (64%) lattice = 1 (1%) other degeneration (cobblestone, naevus, CHRPE, etc. = 26 (34%)	3.87 [1.15–13.0]	0.029
**Type of AMD**	PCV = 38 (50%) others = 38 (50%)	1.05 [0.33–3.38]	0.93
**Contralateral eye at baseline**	no ERM =35 (46%) present = 41 (54%)	1.17 [0.33–4.11]	0.81
**Number of injections**	mean 9.41 (±4.33) (times)	1.02 [0.90–1.16]	0.72
Forward-backwards stepwise method			
**PVD status**	no PVD = 4 (5%) complete PVD = 46 (61%) VMA = 18 (24%) progress = 8 (11%)	5.38 [1.73–16.7]	0.004
**Peripheral degeneration**	none = 49 (64%) lattice = 1 (1%) other degeneration (cobblestone, nevus, CHRPE, etc. = 26 (34%)	3.83 [1.23–12.0]	0.021

*ERM* epiretinal membrane, *OR* odds ratio, *CI* confidence interval, *PVD* posterior vitreous detachment, *VMA* vitreomacular adhesion, *CHRPE* congenital hypertrophy of the retinal pigment epithelium, *AMD* age-related macular degeneration

Table 4 shows another result of the binary logistic regression analysis, detailing aflibercept and ranibizumab independently. The status of PVD and peripheral retinal degeneration were also selected as significant variables throughout forced enter method and forward-backwards stepwise selection analysis and these results corresponded with observations tabulated in Table 3. The number of aflibercept and ranibizumab had negative correlation (*r* = − 0.49, [95% confidence interval (CI): − 0.64 to − 0.29], p<0.0001).

**Table 4:** Multivariate logistic regression analysis about factors that could have influence the appearance or progression of ERMs, examining aflibercept and ranibizumab independently

Variables	OR[95% CI]	p
Forced enter method		
**Smoking**	0.95 [0.26–3.41]	0.94
**Lens status**	1.17 [0.30–4.53]	0.82
**Subretinal hemorrhage**	0.44 [0.04–4.82]	0.50
**PVD status**	5.88 [1.73–20.0]	0.005
**Peripheral degeneration**	3.74 [1.11–12.7]	0.034
**Type of AMD**	1.23 [0.34–4.48]	0.75
**Contralateral eye at baseline**	1.18 [0.33–4.29]	0.80
**Number of aflibercept (mean7.19 ± 4.75)**	1.01 [0.88–1.15]	0.94
**Number of ranibizumab (mean 2.41 ± 3.12)**	0.94 [0.76–1.16]	0.57
Forward-backwards stepwise method		
**PVD status**	5.39 [1.73–16.7]	0.004
**Peripheral degeneration**	3.83 [1.23–12.0]	0.021

Note: number of aflibercept and ranibizumab had negative correlation

*ERM* epiretinal membrane, *OR* odds ratio, *CI* confidence interval, *PVD* posterior vitreous detachment, *AMD* age-related macular degeneration;

(*r* = −0.49, [95%CI -0.64--0.29] *p* < 0.0001, Pearson correlation)

## Discussion

Coexistence of ERMs was reported to be 15–38% of ARMD eyes [3, 4, 24, 25]. The higher prevalence of ERMs with eyes with ARMD than normal eyes adjusted for age was considered to be caused by inflammation or preretinal glial cells found more frequently in ARMD than control [26]. In our results, 61% of ARMD eyes at baseline and 78% of those after 3 years of treatment had presented any ERMs and these were much higher than the existing reports. Previous studies had no strict definition of length of ERMs. The higher prevalence was considered to be caused by criteria used in the form of the minimum region of ERMs in this study.

The reported incidence of VMA in ARMD eyes in literature ranged from 12 to 41% [21, 22, 25, 27], and the higher prevalence than that in controls with adjusted age was considered to be a pathogenesis of ARMD, as traction would lead to pigment epithelial detachment and spread of VEGF [28] . In our results, 61% of eyes with ARMD had complete PVD, 24% of eyes presented with VMA, 5% had no PVD, and 11% had progressed to PVD during 3 years, and were in keeping with the reports discussed.

Cho et al. stated no spontaneous resolution of ERMs in ARMD patients in their study [3] and there were no other reports, to the best of our knowledge, about appearance, progression, or resolution of ERMs in ARMD. The present study is the first report to investigate appearance or progression of ERMs in ARMD patients and evaluate the influences of injection. Regarding other reports among eyes without ARMD, Byon et al. reported that progression from GA to PA was observed in 33% of eyes of idiopathic ERM during 24 months [18]. In our study, GA to PA progression was observed in 24% (8/33) during 36 months.

Regarding the reports [16, 29, 30], which had stated that PVD was significantly associated with the formation of ERMs, Ota et al. reported that idiopathic ERMs with partial PVD had worse visual prognosis than with no PVD or with complete PVD. They suggested that the chronic vitreous traction caused the migration of glial cells, macrophages, or pigment epithelial cells [16]. It led that ARMD itself would have nature in that ERM likely progress throughout higher prevalence of VMA. Moreover, there was a possibility that injection would evoke PVD, though Veloso et al. reported only 7/125 (5.6%) eyes with VMA newly developed PVD after 5 years of treatment with injections [27]. In this study, the proportion of developed PVD during 36 months was 8/76 (11%) and was slightly higher. Therefore, several factors would work together as progressive factors for ERMs in ARMD patients.

The results of the logistic regression analysis, among considerable determining factors, revealed that presence of VMA or progression of PVD during 36 months against no PVD or complete PVD was significantly associated with ERM appearance or progression [OR, 5.77; 95% CI, 1.72–19.4; *p* = 0.005]. Moreover, the presence of peripheral retinal degeneration was significantly associated [OR, 3.87; 95% CI, 1.15–13.0; *p* = 0.029]. The other factors examined were not significantly associated.

As referred previously, incomplete PVD had been considered to progress ERMs with chronic traction [16]. We considered that progression of PVD could also cause rapid migration of glial cells, macrophages, or pigment epithelial cells.

We carefully excluded eyes with a retinal break to prevent contamination of ERMs secondary to the break, and most of the peripheral retinal degeneration prevalence was not due to lattice degeneration (1 eye), but due to cobblestone, naevus, or CHRPE, etc. We could search only one article that reported the prevalence of peripheral retinal degenerations of idiopathic ERMs was similar to that of normal eyes [31]. However, another article reported a higher prevalence in ARMD than in controls [32].

We hypothesized that these peripheral retinal degenerations and vitreoretinal adhesions would co-exist and, vary on the stage or prevalence to some extent, and that both of them are related to the development of ERMs and pathogenesis of ARMD.

There would be possibility that the appearance and progression were affected by both similar factor and different factor. Perhaps, appearance would be evoked by any dynamic change inside the eye ball while progression would reflect a secondary or continuous condition. However, we were unable to conduct an analysis distinguishing appearance and progression owing to the small sample size in this study.

Regarding the present results, there seemed to be no significant influence of the type of AMD, PCV against other groups, on appearance or progression. However, tAMD was reported to have more subjects with incomplete PVD than PCV [21]. If examined with a larger sample size, there might be a significant difference caused by the different status of the PVD.

Exposure to cigarette smoke extracts had been reported to provoke activation of the TGF-β pathway and up-regulate genes related to fibrosis, that was known to play a critical role in the pathogenesis of ERMs [13, 14]. However, the reported results of epidemiologic studies of the relationship between smoking and risk of ERMs were unexpectedly protective and our results had also weak tendency of decreasing odds ratio [33].

Moreover, there was a limited relationship between the history of subretinal hemorrhage and the development of ERMs. We had started this study with the impression that there might be a relationship between developing ERMs in ARMD patients and subretinal hemorrhage treated with aflibercept. However, this hypothesis seemed to be contradicted by the observations. Subretinal hemorrhage inside the retina itself would be unable to cause the development of ERMs, whereas ERMs following vitreous hemorrhage had been distinguished as secondary ERMs [19].

The BMES II study revealed the rate of new appearance of ERMs to be 9.1% in eyes with history of cataract surgery during 5 years from BMES I, whereas it was 4.9% in non-surgical group [15]. In the present study, only one of the 30 eyes that had no ERMs at baseline had undergone cataract surgery during 36 months; therefore, there was no significant influence on the results. The only eye had the new appearance of ERMs.

In the BMESIIstudy, moreover, 13.5% of another eye of the first eye with ERMs at baseline had reported developing new membranes, while 5.3% of another eye of the first eye without ERMs [15]. In our study, these proportions were 60 and 40%, not significantly different. Our high prevalence of ERMs would influence this difference of results.

The observation that the number of injections did not have an impact on the appearance or progression of ERMs could suggest the absence of side effect of anti- VEGF injection in the development of ERMs whereas we had no controls of ARMD eyes that had not received any injections. The fact that the proportion of GA to PA was 24% during 36 months and had no priority to 33% during 24 months of idiopathic ERMs [18] also supports this possibility. Moreover, further comparison of each injection times of aflibercept and ranibizumab independently had no impact on the development of ERMS.

We hypothesize that NO would decrease due to anti-VEGF injection and PDGF-A, TNF-α, TGF-β and some other cytokines would be up-regulated [7–9], however, the changes would be weak due to the dose or present time or these cytokines were unable to develop ERMs by themselves without migration of glial cells, macrophages, or pigment epithelial cells [16], caused by vitreous change. Though there were no further investigations about developing ERMs by injections at BRVO from Marticorena et al. report [6], further prospective studies would be necessary to explain the pathogenesis of secondary ERMs in both BRVO and ARMD.

This study had several limitations. First, there was no control of eyes with ARMD and without any injection, previously referred. Second, this was a retrospective study and invitation spans had variety. Moreover, appearance and progression of the ERMs were not differentiated. A further prospective study differentiating these parameters is necessary.

## Conclusion

The number of anti-VEGF injections in ARMD had little relationship with developing secondary ERMs with neither aflibercept nor ranibizumab and this related possibility that injections alone were unable to develop ERMs. Though their side effects of hypertension or thrombosis following reduction of NO, endophthalmitis, retinal detachment, or iatrogenic cataract are sometimes critical, and clinicians must be aware of these risks. VMA or developing PVD and peripheral retinal degenerations had an association with developing ERMs, and these conditions could be related to the pathogenesis of ARMD.

## Data Availability

The datasets generated and analyzed during the current study are not publicly available because we are not able to permit any possibility of identifying persons from treatment history regardless of data anonymity, but data are available from the corresponding author upon reasonable request.

## Abbreviations

ARMD: age-related macular degeneration
BMES: The Blue Mountains Eye Study
BRVO: branch retinal vein occlusion
CHRPE: congenital hypertrophy of the retinal pigment epithelium
CI: confidence interval
CMR: cellophane macular reflex
ERMs: epiretinal membranes
FA: fluorescein angiography
GA: global adherent (or attachment)
IA: indocyanine green angiography
IRB: institutional review board
Log MAR: logarithm of the minimal angle of resolution
NO: nitric oxide
OCT: optical coherence tomography
OR: Odds ratio
PA: partial adherent (or attachment)
PDGF: Platelet derived growth factor
PMF: preretinal macular fibrosis
PVD: posterior vitreous detachment
RAP: retinal angiomatous proliferation
SD: spectral-domain
TNF: tumor necrosis factor
TGF: transforming growth factor
VA: visual acuity
VEGF: vascular endothelial growth factor
VMA: vitreomacular adhesion
